# Supplementary material for: Age, Dose, and Locomotion: Decoding Vulnerability to Ketamine in C57BL/6J and BALB/c Mice
Source: Biomedicines. 2023 Jun 25;11(7):1821. doi: 10.3390/biomedicines11071821 (PMC10376483; doi:10.3390/biomedicines11071821)
Supplement: Supplementary file 1 [file biomedicines-11-01821-s001.zip › biomedicines-2282795-supplementary.pdf]

Supplementary Files

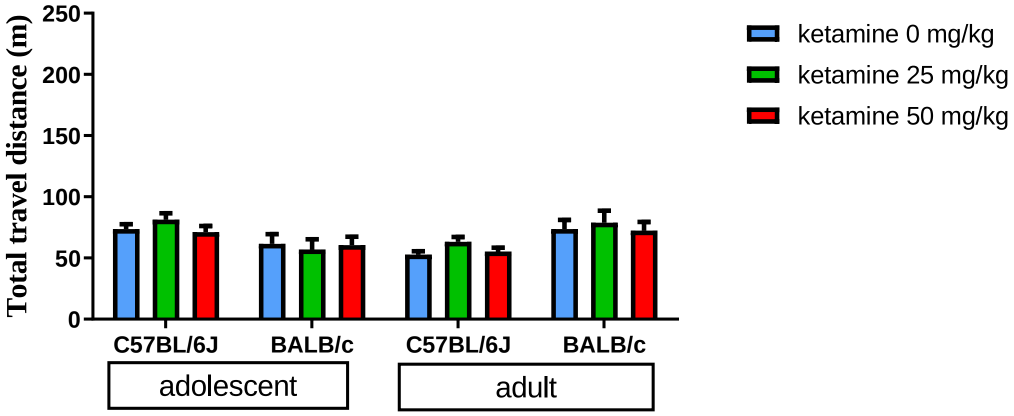

Figure S1: Comparisons of total travelled distance (m) during the habituation period between C57BL/6J and BALB/c treated with 0, 25, and 50 mg/kg of ketamine. The differences of locomotor activity in the open field during first 30 minutes before drug treatment were measured. Vertical bars represent SEM. Differences among the study groups were evaluated by ANOVA and Bon-ferroni's post hoc tests. According to the ANOVA data, there is no significant difference in the total travelled distance during the 30-minute habituation period between the two strain groups (F (1, 126) = 0.084, $p = 0.77$).

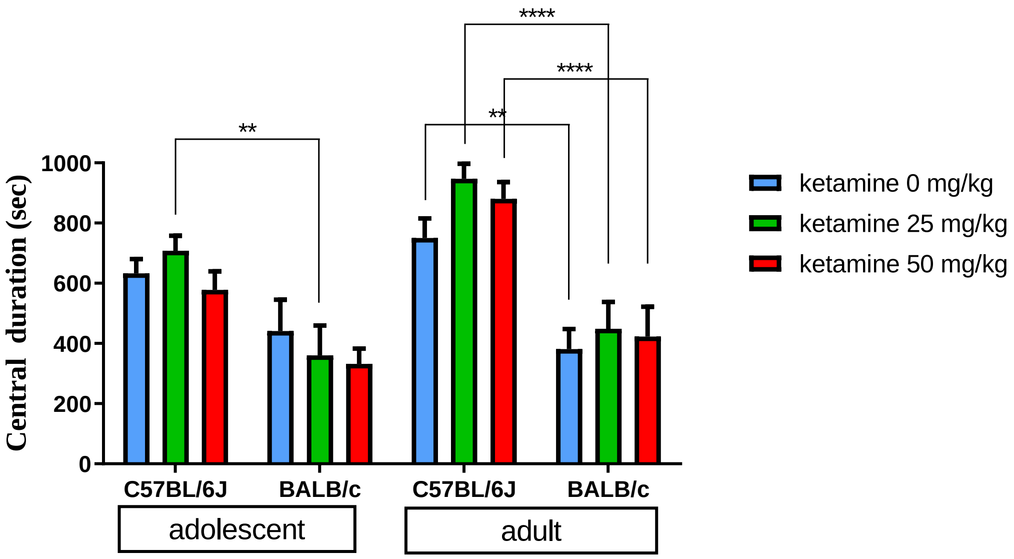

Figure S2: Comparisons of total central duration (sec) during the habituation period between C57BL/6J and BALB/c treated with 0, 25, and 50 mg/kg of ketamine. The differences in time spent in the central area of the open field in first 30 minutes before drug treatment were measured. Vertical bars represent SEM. Differences among the study groups were evaluated by ANOVA and Bonferroni's post hoc tests. According to the ANOVA data, there is a significant difference in the amount of time spent in the central area of the open field during the 30-minute habituation period between the two strain groups (F (1, 126) = 69.36, $p < 0.0001$). Statistically significant differences between groups: $^{**}p < 0.01$, $^{****}p < 0.0001$ vs. C57BL6/J.
